# Nonlinear Dynamics of TNFR1 and TNFR2 Expression on Immune Cells: Genetic and Age-Related Aspects of Inflamm-Aging Mechanisms

**DOI:** 10.3390/biomedicines13040852

**Published:** 2025-04-02

**Authors:** Alina Alshevskaya, Julia Zhukova, Julia Lopatnikova, Filipp Vasilyev, Ivan Khutornoy, Elena Golikova, Fedor Kireev, Sergey Sennikov

**Affiliations:** 1 Federal State Autonomous Educational Institution of Higher Education I.M. Sechenov First Moscow State Medical University of the Ministry of Health of the Russian Federation (Sechenov University), Moscow 119048, Russia; alkkina@yandex.ru (A.A.); lopatnikova18@yandex.ru (J.L.); tamah@inbox.ru (E.G.); 2 Federal State Budgetary Scientific Institution Research Institute of Fundamental and Clinical Immunology, Novosibirsk 630099, Russia; zhukova1982@rambler.ru (J.Z.); vasilyevmd@gmail.com (F.V.); f.kireev@mail.ru (F.K.); 3 Institute of Medicine, Ammosov North-Eastern Federal University in Yakutsk, Yakutsk 677013, Russia; 4 Lomonosov Moscow State University, Moscow 119991, Russia; mcdm.avk@gmail.com

**Keywords:** TNFR1, TNFR2, age-related receptor expression, immune cell subsets, TNF signaling, genetic polymorphisms, immunosenescence, inflamm-aging

## Abstract

**Introduction:** Immunosenescence alters TNF receptor expression (TNFR1 and TNFR2), contributing to chronic inflammation (inflamm-aging) and age-related diseases. Polymorphisms in TNFRSF1A and TNFRSF1B may influence receptor expression; however, their role in age-dependent modulation remains unclear. This study examines TNFR1/TNFR2 expression dynamics on T cells, B cells, and monocytes across different ages and evaluates the impact of genetic polymorphisms. **Methods:** PBMCs from 150 donors (18–60 years) were isolated via density-gradient centrifugation and cultured under spontaneous and LPS-stimulated conditions. TNFR1 and TNFR2 expression on immune cell subsets was quantified using flow cytometry with BD QuantiBRITE PE beads. SNP genotyping in TNFRSF1A and TNFRSF1B was performed via PCR with restriction analysis. Nonlinear age-related trends were assessed using polynomial approximation and inflection point analysis (Tukey’s method). **Results:** Among the 23 analyzed TNF system parameters, the proportion of TNFR2^+^CD3^+^ T cells increased with age, whereas TNFR1^+^ and TNFR2^+^ monocyte populations showed significant negative correlations (*p* < 0.05). Inflection points (~27, 34–36, and 44–45 years) indicated nonlinear dynamics in TNFRs expression during aging. TNFR2 expression on T cells gradually increased and stabilized at later ages, whereas TNFR1 and TNFR2 expression on monocytes followed distinct declining trajectories. Genetic polymorphisms influenced correlation strength, but did not alter direction, demonstrating a conserved pattern of age-related receptor expression shifts. **Conclusions:** TNFR expression exhibits nonlinear, age-dependent alterations across immune cells, shaped by immunosenescence and genetic variability. The identified critical age intervals represent key phases of immune remodeling, where assessing TNFR expression may provide insights into inflamm-aging mechanisms and potential targets for immune modulation.

## 1. Introduction

Aging is associated with profound changes in the immune system, leading to reduced functional capacity and impaired homeostasis [1]. This process, known as immunosenescence, affects both adaptive and innate immunity [2]. One of its key consequences is the increase in chronic low-grade inflammation, termed “inflamm-aging” [3,4]. Among the major regulators of this process is tumor necrosis factor (TNF), a cytokine with pleiotropic effects on immune homeostasis and inflammation [5]. TNF plays a central role in orchestrating immune responses by interacting with two receptors, TNFR1 and TNFR2 [6,7]. Changes in TNF level and its receptor expression are linked to increased susceptibility to infections, reduced vaccine efficacy, and the development of age-related diseases such as atherosclerosis, diabetes, and cancer [8]. While TNFR1 primarily mediates pro-inflammatory and pro-apoptotic signaling, TNFR2 is predominantly associated with anti-inflammatory and regenerative functions. Importantly, these receptors exist in both membrane-bound and soluble forms, which may differentially contribute to immune regulation during aging [9]. An age-related imbalance between TNFR1 and TNFR2 activation may contribute to the progression of inflamm-aging, highlighting TNF signaling as a key target for aging research. Understanding these alterations in TNF signaling may help identify potential therapeutic targets for modulating age-associated immune dysregulation. This is particularly relevant for developing strategies aimed at improving immune resilience and reducing chronic inflammation in aging populations.

Age-related changes impact both adaptive and innate immunity. T lymphocytes exhibit reduced proliferative capacity and altered cytokine profiles. They also accumulate senescent cells, leading to functional dysregulation [10]. Similarly, innate immunity undergoes significant alterations. Monocytes, key players in inflammatory responses, show age-related shifts in functional activity. These changes include modifications in phagocytic capacity and activation marker expression [11]. These processes are central to the development of inflamm-aging and predispose individuals to chronic inflammatory diseases.

In addition to receptor density and distribution, TNFR structures may undergo age-related modifications. Structural changes upon ligand binding or post-translational modifications may influence receptor affinity and downstream signaling efficiency. As a result, immune responses may be altered with age [12]. Beyond age-related alterations, genetic factors play a crucial role. Polymorphisms in genes encoding TNF and its receptors (TNFR1 and TNFR2) are associated with variations in their expression and function, as well as susceptibility to inflammatory and autoimmune diseases [13,14,15]. These genetic variations may either exacerbate or mitigate the effects of aging on TNF receptor expression. This underscores the need for further investigation into their precise role in immune aging.

Despite extensive research on inflamm-aging and TNF signaling [16], there are still limited data on how TNFR1 and TNFR2 expression changes with age across different immune cell populations [17]. Moreover, the relative contributions of membrane-bound versus soluble TNFR1 and TNFR2 signaling in different immune cell subsets remain insufficiently characterized in the context of aging. Filling these knowledge gaps is particularly relevant for developing personalized medicine approaches aimed at correcting immune dysfunctions in aging individuals.

Thus, our study aims to investigate age-related changes in TNFR1 and TNFR2 expression in immune cells, including T lymphocytes, B lymphocytes, and monocytes. We also assess the influence of genetic polymorphisms on these changes. The findings will provide deeper insights into the mechanisms underlying age-related immune alterations. Moreover, given the interindividual variability in TNFR expression and function, these findings may contribute to the development of precision medicine approaches tailored to immune aging. Identifying specific TNFR-related biomarkers could enable more personalized strategies for predicting and mitigating age-related immune dysfunction. They may also help clarify the role of TNF receptors in inflamm-aging and age-associated diseases.

## 2. Methods

### 2.1. Peripheral Blood Mononuclear Cell (PBMC) Isolation and Cultivation

This study was conducted on human peripheral blood mononuclear cells (PBMCs). Whole blood samples were obtained from Blood Procurement Center No. 1 of the Novosibirsk Blood Center, with a cohort consisting of 150 residents of Novosibirsk (83 (55.3%) males, 67 (44.7%) females), aged 18–59 years who provided their written informed consent to participate the study. The research was performed in accordance with the Declaration of Helsinki and was approved by the local ethics committee of the FSBI ‘Research Institute of Clinical Immunology’ (protocol no. 17-14). Although the study included both male and female participants, no sex-stratified analysis was performed, as the sample size was not designed to detect sex-based differences in TNFR expression. Given that immune responses may vary between males and females due to hormonal influences, including menstrual cycle phases, perimenopause, and menopause, future studies with larger, sex-stratified cohorts would be required to explore these effects in detail.

Venous blood was collected under fasting conditions from the cubital vein using sterile 9 mL vacuum tubes containing K_3_-EDTA anticoagulant (tripotassium ethylenediaminetetraacetic acid; Vacuette K_3_-EDTA, Greiner Bio-One GmbH, Frickenhausen, Germany). PBMCs were isolated following a density gradient centrifugation protocol (Böyum, 1968, [18]). Briefly, 4.5 mL of blood, diluted 1:1 with RPMI-1640 medium (BioloT, Saint Petersburg, Russia), was layered onto 3 mL of Ficoll–Urografin (ρ = 1.077 g/L), composed of Ficoll (Pharmacia Fine Chemicals, Stockholm, Sweden) and Urografin (Schering AG, Berlin, Germany). The samples were centrifuged at 1500 rpm for 45 min. PBMCs were collected from the interface and washed twice in RPMI-1640 medium by resuspension followed by centrifugation at 1500 rpm for 10 min, with supernatant removal. The final cell pellet was resuspended and diluted in complete RPMI-1640 medium containing 10% fetal calf serum (FCS) (HyClone, Logan, UT, USA), 2 mM L-glutamine (BioloT, Saint Petersburg, Russia), 5 × 10^−4^ M 2-mercaptoethanol (Sigma-Aldrich, Saint Louis, MO, USA), 80 µg/mL gentamicin (KRKA, Novo mesto, Slovenia), 10 mM HEPES buffer (Sigma-Aldrich, Saint Louis, MO, USA), 100 µg/mL benzylpenicillin (Biosintez, Penza, Russia). The final cell concentration was adjusted to 2 × 10^6^ cells/mL. PBMCs were cultured at a final concentration of 2 × 10^6^ cells/mL in 96-well plates (TPP, Trasadingen, Switzerland) for 24 h in a humidified incubator at 37 °C, 5% CO_2_, either unstimulated or in the presence of 200 ng/mL *Escherichia coli* lipopolysaccharide (LPS, serotype 055:B5, Sigma-Aldrich, Saint Louis, MO, USA).

### 2.2. Assessment of TNF Receptor Expression on PBMC Subpopulations

Phenotypic characterization of immune cell subsets was performed using flow cytometry (FACSAria and FACSVerse, BD Biosciences, Franklin Lakes, NJ, USA) with monoclonal antibodies: APC-conjugated anti-CD3 (clone OKT3), FITC-conjugated anti-CD14 (clone 61D3), and PE-Cy7-conjugated anti-CD19 (clone HIB19) (eBioscience, San Diego, CA, USA), as well as anti-human TNFR1 and anti-human TNFR2 (R&D Systems, Minneapolis, MN, USA). Data acquisition and fluorescence intensity calculations were performed using FacsDiva software ver. 6.1.3. (BD Biosciences, Franklin Lakes, NJ, USA).

To quantify receptor expression, fluorescence intensity values were converted into absolute receptor counts using the BD QuantiBRITE PE kit (BD Biosciences, Franklin Lakes, NJ, USA). This kit contains four fractions of lyophilized beads, each with a defined level of phycoerythrin (PE) molecules. The calibration beads were resuspended in 500 µL of PBS (137 mM NaCl, 2.68 mM KCl, 10 mM Na_2_HPO_4_ × 12H_2_O, 1.47 mM KH_2_PO_4_, 0.53 mM EDTA, and 0.1% NaN_3_), vortexed for 1 min, and analyzed by flow cytometry. Bead populations were gated on the FSC-A/SSC-A dot plot, and 10,000 events were recorded. PE fluorescence histograms were used to place markers at the four calibration peaks (low, medium low, medium high, and high). Following the manufacturer’s instructions, a log–log regression plot was generated to establish a mathematical relationship between fluorescence intensity and the number of PE molecules. This calibration curve was used to convert PE fluorescence intensity into absolute receptor counts per cell for each immune subpopulation. The final receptor density was determined for CD3^+^, CD19^+^, and CD14^+^ cells [14]. TNF receptor expression analysis was performed using the same photomultiplier voltage settings for the PE detector as those used for bead calibration, ensuring accurate conversion of fluorescence intensity into PE molecule counts per cell. These values were further converted into antibody molecules per cell using a 1:1 PE-to-antibody ratio. Flow cytometer performance was monitored weekly using Cytometer Setup and Tracking (CS&T) beads (BD Biosciences, Franklin Lakes, NJ, USA) to ensure consistency.

### 2.3. Genotyping of TNFR1 and TNFR2 Polymorphisms

Genomic DNA was extracted from PBMCs of conditionally healthy individuals using the phenol–chloroform method. Polymorphic variants in the promoter regions of TNFRSF1A and TNFRSF1B genes were genotyped using PCR followed by restriction fragment length polymorphism (RFLP) analysis. The SNPs selected for association analysis with TNF receptor expression levels were retrieved from the NCBI dbSNP database (http://www.ncbi.nlm.nih.gov/snp (accessed on 25 March 2025)) based on their location within promoter regions and high minor allele frequency. Primer sequences for TNFRI-609 G/T (rs4149570), TNFRI -1207 C/G (rs4149569), and TNFRII -1709 A/T (rs652625) were previously published [19,20], while primers for TNFRII -3609 C/T (rs590368) were designed using NCBI Primer-BLAST (http://www.ncbi.nlm.nih.gov/tools/primer-blast (accessed on 25 March 2025)). Specific primers and restriction endonucleases were used for SNP genotyping: TNFRI -609 (rs4149570) was amplified using 5′-CGGACGCTTATCTATATCTC-3′ (forward) and 5′-TTGTAGTCCAGTCACAAGCA-3′ (reverse), followed by digestion with Bst4C I; TNFRI -1207 (rs4149569) was amplified using 5′-TTGGGAGATGTCTGCATCAA-3′ and 5′-TTCTTCGTTTGCTTGTTTTTCA-3′, with restriction by BstC8 I; TNFRII -1709 (rs652625) was genotyped using 5′-GAGTGCTGAGTGAGAAACTG-3′ and 5′-AGCTTGAATTCGTTCCCAGG-3′, followed by digestion with DseD I; and TNFRII-3609 (rs590368) was amplified using 5′-ATGCTTTTGTCCATGCAGGT-3′ and 5′-GCTGTACCCCGTATTAGCTG-3′, with restriction by Msp I. All primers were synthesized by Biosan JSC (Novosibirsk, Russia).

PCR was performed using a PTC-200 DNA Engine amplifier (MJ Research Inc., Watertown, NY, USA). The reaction mixture (20 µL) contained Taq DNA polymerase (SibEnzyme, Novosibirsk, Russia), primers (0.5 µM), deoxynucleoside triphosphates (0.25 mM), genomic DNA (50–200 ng), and buffer. Amplification conditions: 95 °C for 3 min, followed by 30–35 cycles (94 °C—20 s, 58–64 °C—15 s, 72 °C—20 s), with a final extension at 72 °C for 2 min. Amplicons were digested with specific restriction endonucleases (SibEnzyme) at 37–65 °C for 18 h. Fragment analysis was performed using capillary electrophoresis (QIAxcel System, Qiagen, Hilden, Germany) or 2% agarose gel electrophoresis (TAE buffer, 140–150 V, 20–25 min). Visualization was conducted under UV light, and fragment molecular weight was assessed using ImageMaster VDS (Pharmacia Biotech, Uppsala, Sweden).

### 2.4. Statistics

Data collection and analysis were performed using MS Excel 2016 and JASP v.0.16.3 (University of Amsterdam, Amsterdam, Netherlands, 2022). Quantitative data visualization was conducted using raincloud plots. For the analysis of quantitative variables in related groups, the Wilcoxon test or Friedman test was applied, with exact *p*-values reported. Post hoc analyses for the Friedman test were adjusted using Bonferroni and Holm corrections. Results were considered statistically significant at *p* ≤ 0.05. Data visualization, including polynomial regression curve fitting, identification of the point of maximum curvature using Tukey’s method of bends, scatter plots, and heatmaps, was performed using Python (version 3.11) with the following libraries: Matplotlib (version 3.7.1) for plotting, Seaborn (version 0.12.2) for heatmaps, Pandas (version 1.5.3) for data handling, and NumPy (version 1.23.5) for numerical computations. To assess the relationship between immune cell parameters and age, second-order polynomial regression models were applied to each investigated parameter. Polynomial approximations were used to capture the nonlinear relationship between age and TNFR1/TNFR2 expression on CD3^+^ T cells, CD19^+^ B cells, and CD14^+^ monocytes. Age-dependent trends were visualized using regression plots, and inflection points (regions of maximal curvature in the fitted polynomial functions) were identified to characterize critical transition ages in TNF receptor expression dynamics.

## 3. Results

### 3.1. Association Between TNF Receptor Expression and Age Across Genetic Polymorphisms

**Figure 1:**
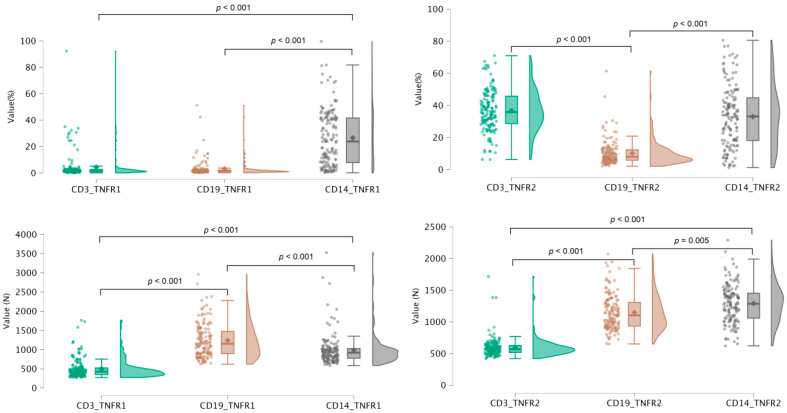
Distribution of TNFR1^+^ and TNFR2^+^ cell proportions among major PBMC subsets in healthy donors, alongside the mean receptor density per expressing cell.

**Figure 2:**
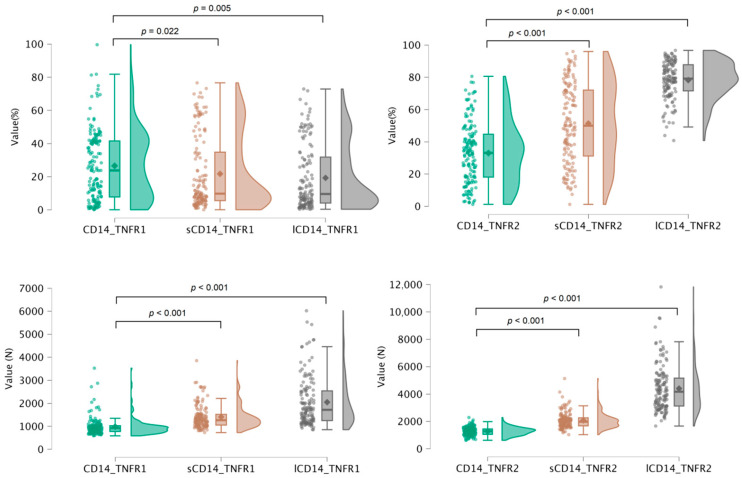
Distribution of TNFR1^+^ and TNFR2^+^ cell proportions among intact CD14^+^ monocytes, spontaneously cultured monocytes, and LPS-stimulated monocytes, alongside the mean receptor density per expressing cell.

Low cytometry analysis of PBMCs before and after in vitro culture, along with ELISA-based quantification of soluble TNF-related factors, enabled the assessment of 23 parameters characterizing TNF receptor system activity in 150 healthy donors aged 18 to 60 years. These parameters included 20 measures of surface receptor expression across immune cell subsets, as well as three serum levels of soluble receptors and TNF cytokine. For all membrane-bound receptor parameters, we analyzed both the percentage of receptor-expressing cells and the absolute receptor count per cell across major immune cell populations (Figure 1 and Figure 2). The analysis revealed significant differences in receptor distribution among cell subsets, as well as dynamic shifts in receptor expression patterns following in vitro stimulation.

**Figure 3:**
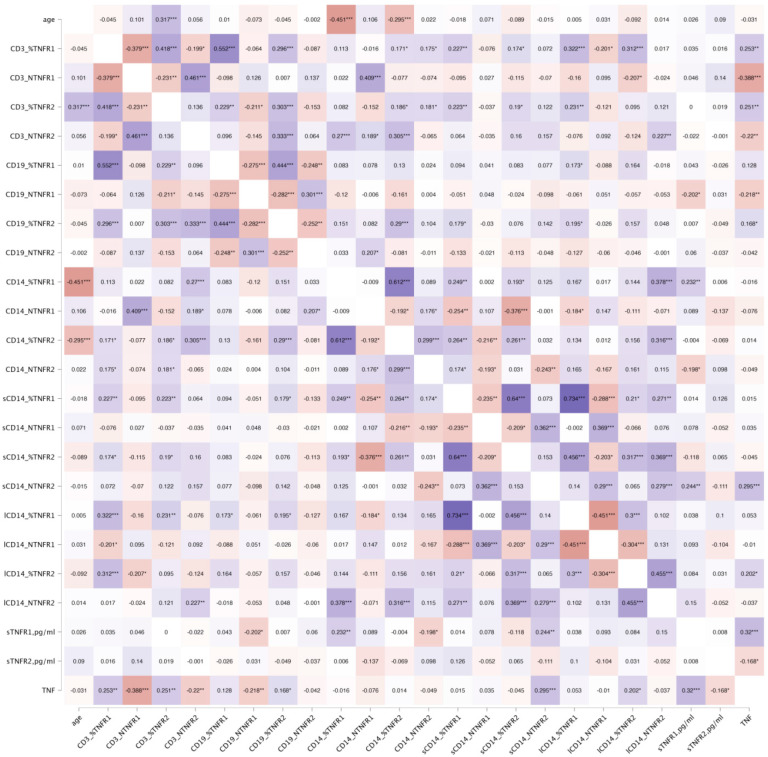
Heatmap of Spearman’s correlation coefficients between age and TNF system parameters, including membrane-bound receptor expression, soluble receptor levels, and serum TNF cytokine concentrations. Statistically significant correlations are indicated with asterisks: * indicates *p* < 0.05, ** indicates *p* < 0.01, *** indicates *p* < 0.001.

**Figure 4:**
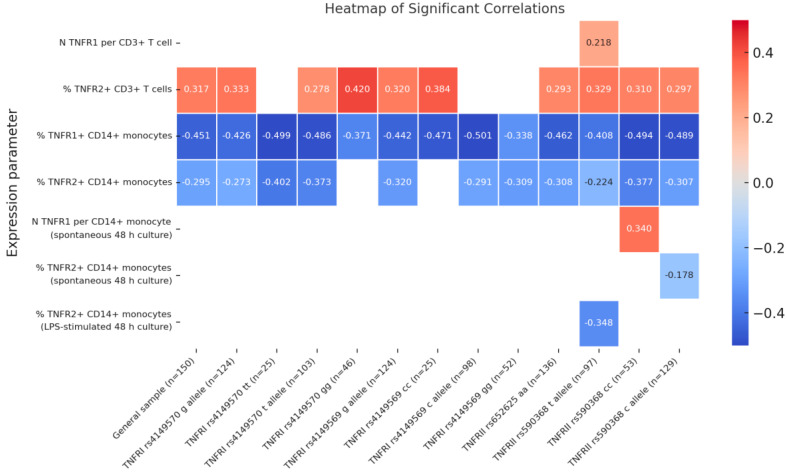
Heatmap of Spearman’s correlation coefficients between age and various TNF system parameters, including membrane-bound receptor expression, soluble receptor levels, and serum TNF cytokine concentrations, in subgroups stratified by genetic polymorphisms. Numerical values represent correlation coefficients, with statistically significant correlations (*p* < 0.05).

To examine age-related associations, Spearman’s rank correlation test was applied to 23 TNFR-related immune parameters in the total donor cohort (Figure 3) and in genetically stratified subgroups (Figure 4). For each of the four TNF receptor system genes, we assessed the contribution of both alleles individually and homozygous genotypes, provided that at least 20 donors carried a given variant. The strongest age-related associations were observed for three TNF receptor expression parameters: the proportion of TNFR2^+^ cells among CD3^+^ T lymphocytes, the proportion of TNFR1^+^ cells among CD14^+^ monocytes, and the proportion of TNFR2^+^ cells among CD14^+^ monocytes. While the correlation strength and significance varied across genetic subgroups, the direction of correlation remained unchanged, indicating a conserved age-related pattern of TNF receptor expression regulation.

In the general cohort, a positive correlation was observed between age and the percentage of TNFR2-positive CD3+ T cells (ρ = 0.317). In carriers of TNFRI rs4149570 g and TNFRI rs4149569 g, the correlation coefficients were slightly higher (ρ = 0.333 and r = 0.320, respectively), with the highest value recorded in the TNFRI rs4149570 gg subgroup (ρ = 0.420) (Figure 4). A negative correlation was found between age and the percentage of TNFR1-positive CD14+ monocytes across all subgroups. In the general cohort, the correlation coefficient was r = −0.451. The strongest correlations were observed in the TNFRI rs4149570 tt (ρ = −0.499) and TNFRI rs4149569 gg (ρ = −0.501) subgroups. Among carriers of the TNFRII rs590368 c allele, the correlation was r = −0.494, while in the TNFRII rs590368 cc subgroup, it was r = −0.408. A negative correlation was also found for TNFR2-positive CD14+ monocytes (ρ = −0.295). The correlation was stronger in the TNFRI rs4149570 tt (ρ = −0.402) and TNFRI rs4149569 gg (ρ = −0.309) subgroups. In the TNFRII rs590368 c subgroup, the coefficient was r = −0.377, exceeding that observed in the TNFRII rs590368 cc subgroup (ρ = −0.307). In spontaneous CD14+ monocyte cultures, a positive correlation was found between age and TNFR1 expression per cell in TNFRI rs4149569 c carriers (ρ = 0.340). Conversely, in the TNFRII rs590368 t subgroup, a negative correlation was observed between age and the percentage of TNFR2-positive cells (ρ = −0.178). In LPS-stimulated CD14+ monocyte cultures, the correlation between age and the percentage of TNFR2-positive cells was r = −0.348 in TNFRI rs4149569 g carriers.

### 3.2. Characterization of the Association Types Between TNF Receptor Expression and Age

**Figure 5:**
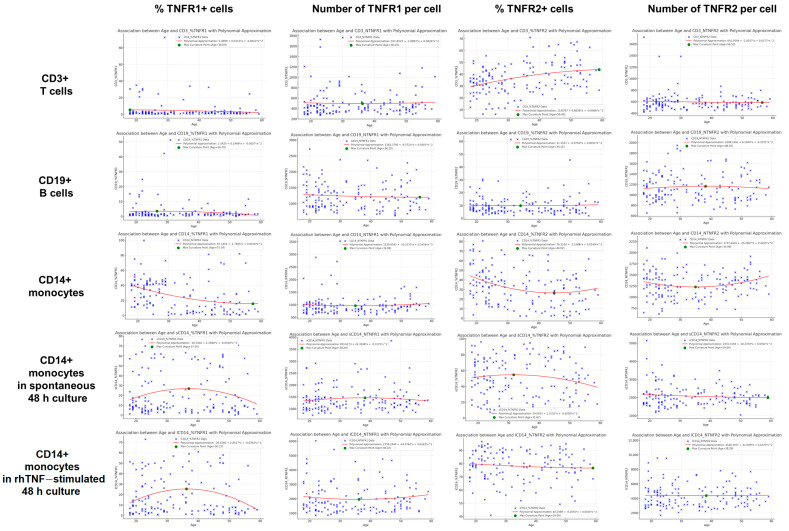
Polynomial approximation (red curve) of the association between age (*x*-axis) and 20 TNF receptor expression parameters (*y*-axis), with inflection points determined using Tukey’s method (marked in green).

Following the identification of significant correlations, we further examined the nature of these associations. Polynomial approximation functions were applied to each association using Tukey’s method of bends, allowing for the identification and validation of inflection points where an increasing trend transitions to a decreasing one (or vice versa) (Figure 5). This analysis revealed distinct trend patterns for all three previously identified statistically significant age-related correlations while also highlighting additional features of age-dependent changes in membrane-bound TNF receptor expression. The proportion of TNFR2^+^ cells among CD3^+^ lymphocytes showed a nonlinear increase with age, stabilizing only at 59 years. The proportion of TNFR1^+^ cells among CD14^+^ monocytes followed an overall declining trend, with a steep decrease at younger ages, followed by a tendency toward stabilization at lower levels in older age groups. However, a precise stabilization threshold could not be identified. In contrast, the proportion of TNFR2^+^ cells among CD14^+^ monocytes demonstrated a U-shaped dependency: it declined from 18 to 44.8 years, reaching a minimum at the inflection point, after which an increasing trend was observed.

**Figure 6:**
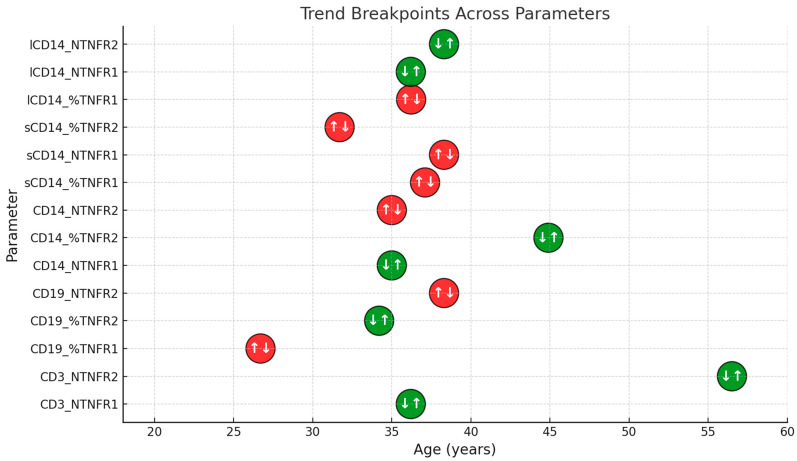
Trend direction of the association between cell percentages and TNFR1/TNFR2 expression levels with donor age, with inflection points calculated using Tukey’s method. Each circle represents a parameter-specific age breakpoint at which the direction of the trend changes. Red circles indicate a switch from an increasing to a decreasing trend (↑↓), whereas green circles indicate a switch from a decreasing to an increasing trend (↓↑). The horizontal axis shows donor age (in years), and the vertical axis lists the analyzed parameters.

Overall, this analysis confirmed the nonlinear nature of age-related immune cell changes, with inflection points varying between 26 and 59 years, depending on the parameter. These findings suggest that different immune cell types and receptors undergo distinct age-related transitions, with specific ages of maximal change identified for each parameter. Notably, some parameters exhibited a shift from growth deceleration to acceleration or from increase to deceleration, underscoring the complexity of immune aging dynamics. These findings highlight the importance of understanding age-related immune system changes for the development of personalized therapeutic strategies. Among the 12 primary parameters assessed in intact immune cells (Figure 5), 7 exhibited inflection points within the central portion of the data range rather than at the extremes (Figure 6). The identified inflection points occurred between 26.7 and 45 years, marking key transition periods in TNF receptor expression trends.

## 4. Discussion

Our results demonstrate a complex, nonlinear age-dependent dynamic of TNFR expression across different immune cell subpopulations. While genetic polymorphisms in TNFR1 and TNFR2 modulate the strength of these associations, they do not alter their direction. We identified critical age intervals (~27, 34–36, and 44–45 years) during which the most pronounced shifts in TNFR-positive cell proportions occur. These trends differ between T lymphocytes and monocytes, highlighting the multifactorial nature of immune regulation during aging.

Notably, our data indicate that aging primarily affects the proportion of TNFR-expressing immune cells rather than receptor density per cell. Such shifts in the distribution of highly and weakly receptor-expressing cell subsets align with previously reported immune system alterations, such as the redistribution of naive and memory T cells [21,22] and the phenomenon of inflamm-aging, characterized by increased levels of pro-inflammatory cytokines [23]. At the same time, several studies suggest that functional changes in receptor signaling pathways (including IL-2/IL-6 receptors and the JAK/STAT cascade) during aging [24] are not necessarily accompanied by significant shifts in absolute receptor counts on the cell surface [25,26]. While the primary focus of this study is the role of TNFR1 and TNFR2 in age-associated immune remodeling, other regulatory mechanisms may also contribute to these changes. Epigenetic modifications, including TNF promoter methylation and histone modifications, have been shown to influence TNF expression with age [27]. Additionally, chronic exposure to pro-inflammatory mediators beyond TNF, such as IL-6, IFN-γ, and IL-1β, may further modulate TNFR signaling and downstream immune responses. These factors, in combination with age-related shifts in metabolic and hormonal regulation, may create a complex network of regulatory influences that shape TNFR expression patterns over time. Further studies are needed to disentangle these interactions and their contributions to immunosenescence. Our findings support the concept that immunosenescence is primarily driven by the redistribution of immune cell subpopulations and their functional state rather than by an obligatory increase or decrease in receptor density per cell.

We identified key TNFR expression parameters that are strongly influenced by age: the most pronounced age-related trends were observed in the proportion of TNFR2^+^ cells among CD3^+^ T lymphocytes and the proportion of TNFR1^+^ and TNFR2^+^ cells among CD14^+^ monocytes. These findings align with previous reports showing that CD3^+^ T lymphocytes progressively lose functional properties with age, become more susceptible to TNF-α–induced apoptotic signaling, and accumulate hallmarks of senescence, including the increase in CD28^−^ subsets and telomere shortening [28,29,30]. Meanwhile, CD14^+^ monocytes in older individuals tend to shift towards a more inflammatory phenotype, likely driven by chronic exposure to pro-inflammatory cytokines and TNF receptor-mediated signaling [31,32]. Collectively, these findings suggest that age-dependent alterations in TNFR expression across immune cell types play a role in either maintaining or disrupting inflammatory homeostasis in aging individuals.

Collectively, these findings suggest that age-dependent alterations in TNFR expression across immune cell types play a role in either maintaining or disrupting inflammatory homeostasis in aging individuals. In addition to their role in immune remodeling, alterations in TNFR expression influence susceptibility to autoimmune diseases and infections in aging individuals. TNFR2 plays a crucial role in immune regulation by supporting the function and stability of regulatory T cells (Tregs), which are essential for maintaining immune tolerance and preventing autoimmunity [33]. A reduction in TNFR2 signaling has been linked to impaired Treg function, contributing to an increased risk of autoimmune diseases such as systemic lupus erythematosus (SLE) and rheumatoid arthritis (RA) [34]. On the other hand, excessive TNFR1 signaling promotes inflammatory responses, exacerbating autoimmune conditions and leading to increased tissue damage [35]. Genetic polymorphisms affecting TNFR1 and TNFR2 have also been associated with altered immune responses, further influencing autoimmune disease susceptibility [36].

Beyond autoimmunity, TNFR alterations also modulate host defense against infections. TNFR1 is essential for initiating effective inflammatory responses to pathogens, promoting cytokine production and immune cell recruitment to infection sites [37]. However, dysregulated TNFR1 signaling can impair pathogen clearance and increase susceptibility to chronic infections [38]. TNFR2, in contrast, plays a more nuanced role by supporting immune cell survival and activation during infections. While TNFR2 signaling enhances protective immunity in certain viral infections, excessive activation may suppress immune responses, increasing vulnerability to opportunistic pathogens.

Furthermore, the clinical use of TNF inhibitors—commonly prescribed for autoimmune disorders—demonstrates the delicate balance of TNFR-mediated immune regulation. By blocking TNF signaling, these therapies can dampen autoimmune activity but also increase the risk of serious infections, such as tuberculosis and pneumonia, due to impaired immune activation [39]. The differential effects of TNF inhibitors on TNFR1 and TNFR2 pathways highlight the complex role of TNFRs in immune homeostasis and pathogen defense.

Taken together, these findings emphasize the dual role of TNFR signaling in aging: on the one hand, supporting immune regulation and tolerance, and on the other, contributing to immune dysregulation and disease susceptibility. Further research is needed to develop targeted interventions that selectively modulate TNFR pathways to enhance immune resilience while minimizing risks of inflammation-driven pathology or immunosuppression.

Interestingly, we found that age-related shifts in the proportion of TNFR-positive cells were independent of genetic polymorphisms in TNFR genes. This is consistent with previous studies showing that while polymorphisms in immune-related genes (e.g., CD14) may influence immune responses and the expression levels of specific markers [40,41], more universal mechanisms govern age-related immune changes. These include epigenetic modifications, such as TNF promoter methylation [42], shifts in naïve-to-effector cell ratios [43,44], and other age-related factors, including hormonal changes and chronic inflammation. Thus, the observed age-dependent increase or decrease in TNFR-positive cell proportions occurs across all genetic TNFR variants, indicating a fundamental and universal role for these processes in immunosenescence.

In the CD3^+^ T lymphocyte population, the proportion of TNFR-positive cells increases with age, whereas in CD14^+^ monocytes, the proportion of TNFR1^+^ and TNFR2^+^ cells decreases. These findings suggest an opposite directional shift in TNFR expression between different immune cell subsets. The age-related increase in TNFR expression among CD3^+^ T cells may reflect the expansion of senescent and effector T-cell subsets, which are more sensitive to pro-inflammatory stimuli, particularly TNF-α [43,45]. The elevated chronic inflammatory state (“inflamm-aging”) may further drive the expansion of TNFR-expressing T cells, reinforcing their role in the regulation of inflammation in aging individuals [46,47].

In contrast, CD14^+^ monocytes exhibit a decline in TNFR-positive cell proportions with age, suggesting a shift toward a more pro-inflammatory CD16^+^ monocyte phenotype, which paradoxically reduces overall sensitivity to TNF signaling [10,31]. It is hypothesized that aged monocytes lose or downregulate the expression of key receptors required for full activation via TNF, a process linked to mitochondrial dysfunction, oxidative stress, and impaired innate immune function [48,49]. A hallmark of monocyte aging is the imbalance in subset distribution, characterized by a decrease in classical monocytes and an expansion of non-classical monocytes, a shift consistently observed in studies of elderly populations [50]. Additionally, monocytes from older individuals exhibit higher baseline cytokine production than their younger counterparts, contributing to the chronic low-grade inflammation known as inflamm-aging [51]. Collectively, these findings suggest that aging leads to a pro-inflammatory, dysfunctional monocyte profile and a loss of plasticity in response to age-related and stimulatory changes. This reduced plasticity may indicate that aged monocytes lose their ability to transition between different functional states (e.g., from activation to regulation), making the organism more susceptible to chronic inflammatory conditions and impairing immune responses to infections [52]. The loss of TNFR2 adaptability may also be linked to the exhaustion of intracellular signaling pathways, leading to a reduced capacity to adjust to inflammatory stimuli [53]. These opposing age-related trends in TNFR expression on T lymphocytes and monocytes highlight the complex and multifaceted nature of immune remodeling during aging.

Our analysis identified several critical age intervals (~27, 34–36, and 44–45 years), during which significant changes in TNFR expression across different immune cell types were observed. In CD3^+^ T-cell-associated parameters, an upward trend was observed after the inflection point (i.e., increased expression of both TNFR1 and TNFR2 beyond a certain age). Conversely, in CD19^+^ B cells and CD14^+^ monocytes, both upward and downward shifts were observed across different parameters. In some cases (e.g., the number of TNFR1 and TNFR2 on CD3+ T cells), a simultaneous increase in both pro-inflammatory and anti-inflammatory receptors was noted, suggesting an adaptive response aimed at balancing inflammatory and regulatory mechanisms during aging. The identification of specific inflection points at ~27, 34–36, and 44–45 years suggests that immune system remodeling does not occur in a linear fashion but rather in discrete transitions, reflecting a combination of physiological and hormonal changes as well as the accumulation of subclinical inflammation. By the late second decade of life (~27 years), immune homeostasis may reach its full maturity, followed by a gradual increase in inflamm-aging [54]. During 34–36 years, some individuals may experience early age-related changes due to shifts in hormonal balance and metabolic regulation, which affect TNF receptor expression via cytokine regulatory networks and senescence-associated mechanisms [55,56]. By 44–45 years, women often undergo perimenopausal transitions accompanied by declining estrogen levels, which can modulate TNFR expression and enhance inflammatory responses [55]. In men, age-related hormonal adjustments, including decreasing testosterone and increasing cortisol levels, may also influence immune regulation. Additionally, by midlife, a pool of senescent cells accumulates, characterized by elevated p16^Ink4a^ expression and increased TNF-α production, leading to critical shifts in cellular sensitivity to inflammatory signals [44]. Thus, these age intervals likely represent key stages in immune system regulation and development, where even minor fluctuations in cytokine and hormone levels can drive substantial shifts in TNFR-positive cell proportions. Taken together, our findings highlight the complex, nonlinear dynamics of TNFR expression during aging, with both increasing and decreasing trends depending on cell type and receptor subtype.

A notable inflection point was observed between 35 and 45 years of age, where both the percentage of TNFR2-positive cells and TNFR2 receptor density on monocytes exhibited an upward trend. This may reflect an age-related enhancement of regulatory activity and compensatory mechanisms via TNFR2, aimed at controlling inflammation [57]. Such dynamics may represent an adaptive response to the increasing inflammatory stimuli associated with aging, highlighting the need to maintain a balance between pro-inflammatory and anti-inflammatory responses.

However, when CD14^+^ monocytes were cultured in vitro, both in spontaneous and LPS-stimulated conditions, a significant shift in TNFR2 expression dynamics was observed. A decline in TNFR2-positive cells, combined with a marked decrease in TNFR1 expression in spontaneous cultures after 32 years of age, suggests that monocytes progressively lose the ability to maintain an anti-inflammatory phenotype under in vitro conditions. This may be due to the exhaustion of regulatory mechanisms or reduced sensitivity to stimulatory factors. In LPS-stimulated cultures, monocytes also demonstrated a loss of capacity to upregulate TNFR2 expression, which may indicate impaired responsiveness to pro-inflammatory stimuli. This is particularly relevant, as LPS is a potent activator of inflammatory responses, and such a decline in reactivity may reflect a functional deterioration of monocytes in inflammatory conditions. Previously described mechanisms support these observations: prolonged LPS stimulation has been shown to induce oxidative and nitrosative stress, leading to DNA damage [58], which in turn activates a senescent phenotype and promotes cell death. Additionally, endotoxin tolerance, characterized by a dramatic reduction in key mediator production and decreased antigen-presenting capacity, has been reported under chronic inflammatory conditions [59]. Moreover, the functional activity of monocytes, including phagocytosis and response to pro-inflammatory cytokines, declines under age-related or stress-induced conditions, thereby impairing the establishment of a robust anti-inflammatory response [60]. Taken together, these factors may contribute to the age-associated decline in monocyte responsiveness to regulatory stimuli in vitro, which is reflected by the reduced TNFR1 expression in older individuals.

The observed dynamics underscore the importance of monitoring TNFR1 and TNFR2 expression changes in monocytes across different age groups, particularly between 35 and 45 years, when the most significant shifts occur. Understanding these changes could help develop targeted therapeutic strategies to preserve immune regulatory function and reduce the risk of chronic inflammatory diseases. Interventions aimed at maintaining or restoring TNFR2 expression may enhance the anti-inflammatory potential of immune cells and restore their ability to respond effectively to inflammatory stimuli, which is particularly relevant for elderly individuals. Overall, our findings demonstrate that age-related alterations in TNFR expression on monocytes play a crucial role in maintaining the balance between pro-inflammatory and regulatory responses, and the loss of this balance may contribute to chronic inflammation and immune dysfunction.

Several parameters, including soluble TNFR1 (sTNFR1) and TNFR2 (sTNFR2) serum levels, as well as TNFR expression density per cell in B lymphocytes, did not demonstrate statistically significant age-related trends. This may be attributed to the influence of additional regulatory factors, such as cytokine-mediated receptor shedding, interindividual variability in immune homeostasis, and compensatory mechanisms that balance receptor expression across different immune cell subsets. These findings indicate that while TNF receptor dynamics exhibit robust age-dependent trends in T cells and monocytes, certain TNF system parameters may be more influenced by short-term inflammatory stimuli rather than long-term age-associated shifts.

This study has some limitations. First, despite the large sample size for flow cytometry studies, the heterogeneity of genetic polymorphisms and the low prevalence of some variants restricted our ability to analyze all potential subgroups. Second, the cross-sectional design (i.e., lack of longitudinal follow-up) limits causal interpretations of age-dependent TNFR expression changes within the same individuals over time. Third, although we included multiple TNFR1/2 gene polymorphisms, not all possible genetic variants and haplotypes were covered. Furthermore, we did not investigate epigenetic regulatory mechanisms, such as TNF promoter methylation, which may significantly influence age-related TNFR expression dynamics.

## 5. Conclusions

Age-related changes in TNFR expression across immune cell types exhibit complex dynamics, with both upregulation and downregulation depending on cell type and receptor subtype. The most pronounced changes occur between 35 and 45 years of age, a period marked by immune system restructuring and shifts in pro-inflammatory and regulatory mechanisms. These findings emphasize the need for age-related monitoring of immune receptor expression to maintain a balance between inflammatory responses and immune regulation. Additionally, they highlight the potential for targeted interventions aimed at preserving normal immune function in aging individuals. Future longitudinal studies incorporating a broader range of immunological and genetic analyses are essential to develop clinical strategies for minimizing the adverse effects of immunosenescence.

## Data Availability

The raw data supporting the conclusions of this article will be made available by the authors on request.
